# The Effects on Bronchial Epithelial Mucociliary Cultures of Coarse, Fine, and Ultrafine Particulate Matter From an Underground Railway Station

**DOI:** 10.1093/toxsci/kfv034

**Published:** 2015-02-10

**Authors:** Matthew Loxham, Rebecca J. Morgan-Walsh, Matthew J. Cooper, Cornelia Blume, Emily J. Swindle, Patrick W. Dennison, Peter H. Howarth, Flemming R. Cassee, Damon A. H. Teagle, Martin R. Palmer, Donna E. Davies

**Affiliations:** *Academic Unit of Clinical and Experimental Sciences, University of Southampton Faculty of Medicine, University Hospital Southampton, Southampton SO16 6YD, United Kingdom, ^†^Institute for Life Sciences, Highfield Campus, University of Southampton, Southampton SO17 1BJ, United Kingdom, ^‡^Ocean and Earth Science, National Oceanography Centre Southampton, University of Southampton, Southampton SO14 3ZH, United Kingdom, ^§^NIHR Southampton Respiratory Biomedical Research Unit, University Hospital Southampton, Southampton SO16 6YD, United Kingdom, ^¶^Centre for Sustainability, Environment, and Health, National Institute for Public Health and the Environment (RIVM), 3720BA Bilthoven, The Netherlands and ^||^Institute for Risk Assessment Sciences (IRAS), Utrecht University, 3508TC Utrecht, The Netherlands

**Keywords:** bronchial epithelium, environmental exposure, metals, particulate matter, primary cell culture, underground railway

## Abstract

We have previously shown that underground railway particulate matter (PM) is rich in iron and other transition metals across coarse (PM_10–2.5_), fine (PM_2.5_), and quasi-ultrafine (PM_0.18_) fractions and is able to generate reactive oxygen species (ROS). However, there is little knowledge of whether the metal-rich nature of such particles exerts toxic effects in mucus-covered airway epithelial cell cultures or whether there is an increased risk posed by the ultrafine fraction. Monolayer and mucociliary air-liquid interface (ALI) cultures of primary bronchial epithelial cells (PBECs) were exposed to size-fractionated underground railway PM (1.1–11.1 µg/cm^2^) and release of lactate dehydrogenase and IL-8 was assayed. ROS generation was measured, and the mechanism of generation studied using desferrioxamine (DFX) and N-acetylcysteine (NAC). Expression of heme oxygenase-1 (HO-1) was determined by RT-qPCR. Particle uptake was studied by transmission electron microscopy. Underground PM increased IL-8 release from PBECs, but this was diminished in mucus-secreting ALI cultures. Fine and ultrafine PM generated a greater level of ROS than coarse PM. ROS generation by ultrafine PM was ameliorated by DFX and NAC, suggesting an iron-dependent mechanism. Despite the presence of mucus, ALI cultures displayed increased HO-1 expression. Intracellular PM was observed within vesicles, mitochondria, and free in the cytosol. The results indicate that, although the mucous layer appears to confer some protection against underground PM, ALI PBECs nonetheless detect PM and mount an antioxidant response. The combination of increased ROS-generating ability of the metal-rich ultrafine fraction and ability of PM to penetrate the mucous layer merits further research.

It has long been known that air pollution can exert harmful effects (Logan, 1953), but it is only relatively recently that exposure to coarse and fine particulate matter (PM) has been linked to mortality from lung cancer and cardiopulmonary disease (Dockery *et al.*, 1993), whereas other studies have looked at the epidemiology of health effects due to pollution from more specific sources (Janssen *et al.*, 2003; Pope, 1989). The next stage is to examine the link between PM source and composition, and their mechanism of action.

Transition metals are of importance in the toxicity of PM due to their ability to act as electron donors, catalyzing reactive oxygen species (ROS) generation in a series of successive one electron reductions, reducing molecular oxygen to superoxide, hydrogen peroxide, and, by the Fenton reaction, the hydroxyl radical (van Klaveren and Nemery, 1999). ROS can react with a range of biological molecules such as DNA, proteins, lipids, and antioxidants and may also perturb nitric oxide signaling (Bowler and Crapo, 2002). As a means of protection against ROS, cells may up-regulate production of antioxidant molecules and enzymes such as heme oxygenase 1 (HO-1) and superoxide dismutase (Li *et al.*, 2004). If increased antioxidant expression is unable to ameliorate the presence of ROS, an inflammatory response may be seen and, with overwhelming ROS attack, cell death may occur (Xiao *et al.*, 2003).

Coarse and fine airborne PM in underground railways contains high levels of transition metals and has been found to be more toxic than particles from other sources (Karlsson *et al.*, 2006; Lindbom *et al.*, 2006), which may be a consequence of its high metal content (Bachoual *et al.*, 2007). Markers of systemic inflammation and procoagulant factors may be raised in the blood of underground railway workers (Bigert *et al.*, 2008), and it has been suggested that asthmatics may show a heightened inflammatory response to underground railway PM (Klepczynska-Nystrom *et al.*, 2012), although there is, as yet, little evidence of effects on lung function (Bigert *et al.*, 2011), myocardial infarction (Bigert *et al.*, 2007), or lung cancer (Gustavsson *et al.*, 2008).

Unlike for coarse and fine PM, there is a paucity of studies which have investigated the composition of ultrafine underground PM and, to the best of our knowledge, none which have examined the potential biological effects of ultrafine underground PM *in vitro*. In many environments, ultrafine PM may account for the majority of particles by particle number, and the aerodynamic diameter <0.1 µm endows them with a high surface area/volume ratio and the potential to translocate from the lungs to the microvasculature (Geiser *et al.*, 2005), and thence to the systemic circulation (Choi *et al.*, 2010). There is thus a need to improve understanding of the risks of ultrafine PM.

The bronchi are lined with a pseudostratified epithelium of ciliated columnar epithelial cells. In addition to providing a selectively permeable, dynamically regulated barrier to the passage of ions, macromolecules, and pathogens, principally by virtue of tight junctions, the epithelium also acts as an immunologic barrier and is able to secrete a battery of cytokines and chemokines to attract and modulate the behavior of a range of immune cells (Loxham *et al.*, 2014). In addition, airway goblet cells secrete mucus, which may trap inhaled toxicants by steric, hydrophobic, and electrostatic interactions (Jachak *et al.*, 2012). As the primary site of deposition of inhaled PM, the airway epithelium is thought to be of crucial importance in understanding the potential effects of such particles.

The aim of this research was to examine the effects on differentiated bronchial epithelial cells of coarse, fine, and ultrafine PM collected from a European underground railway station. The hypothesis being tested was that underground railway PM would elicit a proinflammatory and antioxidant response in a size-dependent manner, by virtue of its iron content.

## MATERIALS AND METHODS

### 

#### 

##### Collection of PM

PM was collected at a European mainline underground railway station as coarse (PM_10__–__2.5_) fine/ultrafine (PM_2.5_; hereafter referred to as “fine”) and quasi-ultrafine (PM_0.18_) suspensions in ultrapure water as detailed previously (Loxham *et al.*, 2013) (see also Supplementary methods). All PM was gamma irradiated (1250 Gy, 10 h) with a Gammacell 1000 cell irradiator (Atomic Energy of Canada Ltd, Ontario, Canada).

##### PM chemical analysis

PM composition was analyzed by inductively coupled plasma mass spectrometry as described previously (Loxham *et al.*, 2013).

##### Particulate lipopolysaccharide concentration

Lipopolysaccharide (LPS) concentrations were determined using a chromogenic Limulus amoebocyte lysate assay (Lonza, Slough, UK) according to the manufacturer’s instructions.

##### Cell culture

Primary bronchial epithelial cells (PBECs) were grown from brushings obtained by fiber optic bronchoscopy as described previously (Bucchieri *et al.*, 2002). Volunteers were classified as severely asthmatic according to the Global Initiative for Asthma Guidelines or nonasthmatic control subjects (donor information in Supplementary table S1). Chronic obstructive pulmonary disease was excluded by spirometry. Prior ethical approval was granted by the Southampton Local Research Ethics Committee (Rec. No. 05/Q1702/165, code MRC0268), and written informed consent was obtained. PBECs or 16HBE cells (a gift from Professor D.C. Gruenert, San Francisco, CA) were cultured as monolayers or at air-liquid interface (ALI) on 0.4-µm pore Transwell inserts as described previously (Leino *et al.*, 2013; Loxham *et al.*, 2013) (see also Supplementary methods). Twenty-four hours prior to challenge, cells were made quiescent in bronchial epithelial basal medium (BEBM) containing 1% 100x insulin/transferrin/selenium solution (Sigma-Aldrich, Gillingham, UK) and either 1 mg/ml bovine serum albumin (BSA; PBEC monolayers) or 1.5 µg/ml BSA (ALI), or 1 mg/ml BSA in MEM with GlutaMax (16HBE) prior to use (Lordan *et al.*, 2002).

##### Challenge of cells with PM

ALIs or 80–90% confluent monolayers were challenged (into the apical compartment for ALI cultures) with PM stock suspensions diluted in serum-free/protein-free medium (see Supplementary methods), supplemented with desferrioxamine (DFX) or N-acetylcysteine (NAC) (both Sigma-Aldrich) where indicated. All ALI culture challenges were performed in 6.5 mm diameter, 0.4-µm pore size collagen-coated polyester Transwell inserts, placed into wells of standard 24-well cell culture plates. All monolayer challenges were performed in standard collagen-coated 24-well plates, except for 2,7-dichlorofluorescein (DCF) experiments, which were performed in equivalent collagen-coated 96-well plates. Volumes used were 75 µl for Transwell (300 µl medium in basolateral compartment) and 96-well assays, and 400 µl for 24-well plates. The resultant supernatants were centrifuged at 16 000 × g for 10 min at 4°C before storage at −80°C.

##### Lactate dehydrogenase release

Lactate dehydrogenase (LDH) release was measured using a CytoTox 96 Nonradioactive Cytotoxicity Assay (Promega, Southampton, UK), according to the manufacturer’s instructions. All samples were assayed in duplicate. Intracellular LDH in control cultures was determined by lysing cells with 1% Triton X-100 in BEBM. LDH release in each well was calculated as a percentage of total LDH, and values for control wells were subtracted from PM-treated wells to give the percentage of total LDH released as a result of PM challenge.

##### IL-8 release

IL-8 concentration of culture supernatants was determined using a human IL-8 DuoSet ELISA kit (R&D Systems, Abingdon, UK), according to the manufacturer’s protocol. All samples were assayed in duplicate.

##### FITC-dextran passage

Macromolecular permeability of the epithelial barrier of ALI cultures was assessed using 4 kDa dextran labeled with fluorescein isothiocyanate (FITC), as described previously (Leino *et al.*, 2013). Briefly, after 24 h challenge with PM, apical supernatant was removed and the apical compartment was washed once with Hanks’ balanced salt solution (HBSS), and the basolateral medium was replaced with 300 µl fresh medium; 100 µl 2 mg/ml 4 kDa FITC-dextran in BEBM was added to the apical compartment, and cultures were incubated for 24 h as previously. Fluorescence of duplicate 100 µl aliquots from each basolateral compartment was then determined at 485 nm and 530 nm excitation and emission wavelengths, respectively, and basolateral FITC-dextran concentration determined using a standard curve with standards prepared and incubated in parallel.

##### Antioxidant gene responses to PM

After incubation with PM, cells were lysed using Trizol lysis reagent (Life Technologies, Paisley, UK), and RNA was extracted using a standard phenol-chloroform extraction protocol. RNA was reverse transcribed to cDNA using a Precision Reverse Transcription kit (PrimerDesign, Southampton, UK) according to the manufacturer’s instructions. Expression of HO-1 and NQO1 was determined using probe-based qPCR assays (PrimerDesign and Applied Biosystems, Paisley, UK, respectively), whereas expression of the housekeeping genes ubiquitin C and glyceraldehyde 3-phosphate dehydrogenase was determined using a probe-based duplex primer mix (PrimerDesign). Fold change in gene expression relative to PM-free, time-matched controls was determined using the ΔΔCt method (see Supplementary data for further details).

##### Measurement of ROS generation

Generation of ROS in PM-challenged cell cultures was determined by use of the dye DCF, which becomes fluorescent upon oxidation, for example, by ROS. DCF (10 µM) was loaded into cells as a diacetate, with the acetyl groups being cleaved by intracellular esterases to achieve intracellular sequestration. After 30 min loading, cells were washed twice with HBSS, before being challenged with underground railway PM. Fluorescence at excitation and emission wavelengths of 485 nm and 530 nm, respectively, was measured at regular intervals.

##### Transmission electron microscopy

ALI cultures were fixed, contrast stained, dehydrated, and embedded in Spurr resin prior to ultrathin sectioning. Sections were viewed on an H7000 transmission electron microscope (Hitachi High-Technologies Europe GmbH, Maidenhead, UK). Energy dispersive x-ray spectroscopy was performed using a Tecnai12 EDAX transmission electron microscope (FEI, Hillsboro, OR).

##### Statistical analysis

Analyses were performed using GraphPad Prism 6 (GraphPad Software, San Diego, CA). Significance between groups was determined by one-way repeated measures ANOVA with Bonferroni correction, or Friedman test with Dunn’s correction, for normally and nonnormally distributed data, respectively. Significance was reached if *P* < 0.05. All data in text are presented as mean ± SEM unless stated otherwise.

## RESULTS

### 

#### 

##### Chemical composition of underground railway PM

Physicochemical analysis of the underground railway PM used in this study has been published previously (Loxham *et al.*, 2013). This study used PM from a single collection day, with airborne PM concentrations of 180 µg/m^3^, 71 µg/m^3^, and 44 µg/m^3^ for coarse, fine, and ultrafine fractions, respectively. PM composition is listed in Table 1. LPS content was 8.5 endotoxin units (EU)/mg (coarse), 5.6 EU/mg (fine), and 6.7 EU/mg (ultrafine).

**TABLE 1.** The Elemental Composition of Coarse, Fine, and Ultrafine Particulate Matter (PM) From an Underground Railway Station

Element	Coarse (mg/g)	Fine (mg/g)	Ultrafine (mg/g)
Fe	321	284	329
Mg	16.3	21.2	25.6
Ca	15.2	15.2	22.0
Cu	16.8	14.1	17.1
Zn	6.16	6.19	8.11
Al	6.13	5.39	6.76
Na	4.05	2.48	4.05
K	2.03	1.54	3.20
Mn	2.88	2.52	2.96
Ba	2.16	2.2	2.67
Sb	1.82	1.84	2.27
Zr	0.41	1.28	1.50
Mo	0.83	1.02	1.08
B	0.42	0.21	1.04
Cr	0.54	0.45	0.51
Ti	0.44	0.37	0.40
Ga	0.21	0.22	0.27
Ni	0.18	0.17	0.19

PM was collected at a busy mainline underground railway station over a 9 h sampling period. The elemental composition of the particulate matter was analyzed by inductively coupled plasma mass spectrometry. Only elements comprising at least 0.1 mg/g in at least one size fraction are listed.

##### IL-8 release by PBEC monolayers in response to underground railway PM

**FIG. 1.**
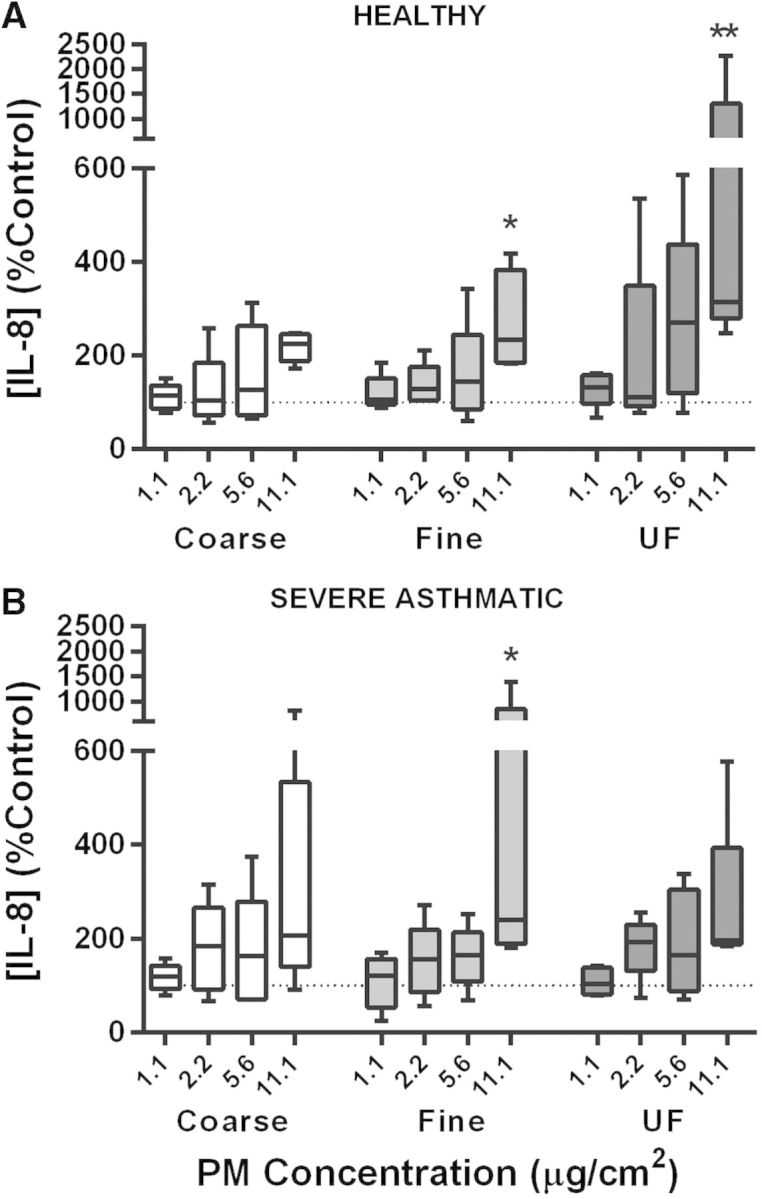
The effect of underground railway particulate matter (PM) on IL-8 release from healthy and severely asthmatic donor primary bronchial epithelial cell (PBEC) monolayers. Epithelial cell monolayers grown from cells of healthy (top) or severely asthmatic (bottom) donors were exposed to varying concentrations of coarse, fine, or ultrafine underground railway PM for 24 h. Basolateral supernatant IL-8 concentration was analyzed by ELISA, with supernatants assayed in duplicate. IL-8 release is expressed as percentage of IL-8 release from cells incubated in control medium for 24 h. Median IL-8 concentration in controls was 732 pg/ml, IQR 475–963 pg/ml (healthy), and 931 pg/ml, IQR 367–1357 pg/ml (severe asthmatic). Results presented as median with 25th and 75th percentile, whiskers represent 10th and 90th percentiles. *P < 0.05 vs. control, **P < 0.01 vs*.* control, *n* = 5 healthy donors, five severely asthmatic donors.

PBEC monolayers were exposed to underground PM (1.1, 2.2, 5.6, 11.1 µg/cm^2^, corresponding to 5, 10, 25, and 50 µg/ml, respectively) for 24 h to characterize basic concentration–response relationships. Healthy donor cultures tended to show increased IL-8 release with increasing PM concentration for all three size fractions, with a further trend toward an increased upper bound of IL-8 release with increased particle concentration suggestive of an increased response among some individuals (Fig. 1). The highest concentrations of fine underground PM (234% of control, interquartile range (IQR): 185–383%, *P* < 0.05) and ultrafine underground PM (315% of control, IQR: 280–1302%, *P* < 0.01) elicited a significant increase compared with control. Conversely, 10 ng/ml TNF-α resulted in an increase of 1453% of control (IQR: 739–1848%) (not shown). A similar trend, but with no significant effect of ultrafine PM, was observed in cultures from severely asthmatic donors, with no significant difference in responses compared with healthy donor cultures and no significant difference in response to TNF-α (*P* = 0.53). The lack of increased LDH release in response to underground PM suggested the absence of significant cell death (Supplementary fig. S1).

##### ROS generation by underground railway PM

**FIG. 2.**
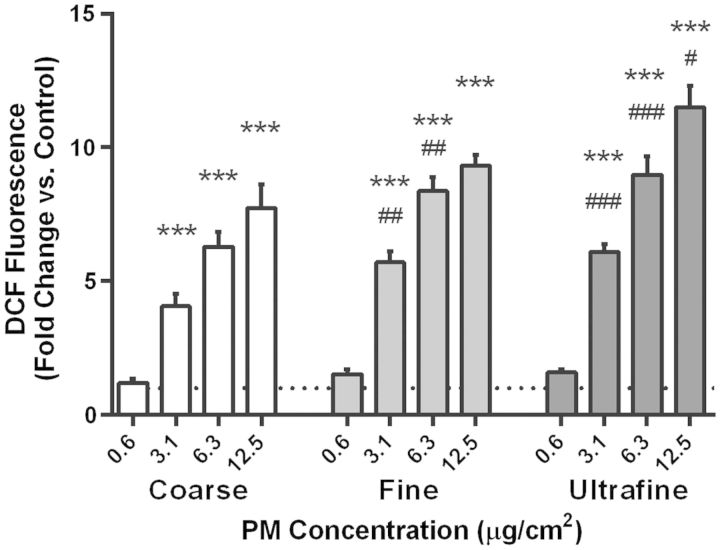
Changes in 2,7-dichlorofluorescein (DCF) fluorescence after 3 h with underground railway particulate matter (PM). Coarse, fine, or ultrafine underground railway PM was applied to primary bronchial epithelial cell (PBEC) monolayers preloaded with DCF. After 3 h, fluorescence of the well was measured (excitation 485 nm/emission 530 nm). Data were calculated as mean fluorescence intensity of PM-challenged wells divided by mean fluorescence intensity of cells exposed to PBS-supplemented culture medium, all in duplicate. Mean control DCF fluorescence was 1.7 ± 0.1 relative fluorescence units (RFU). ***P < 0.001 vs*.* control. #*P* < 0.05, ##*P* < 0.01, ###*P* < 0.001 vs*.* respective concentration of coarse PM, *n* = 3–5 healthy donors.

PBEC monolayers were loaded with the ROS-sensitive dye DCF, and fluorescence was determined relative to PM-free controls after 3 h exposure to underground PM (Fig. 2). For each size fraction of PM, there was a concentration-dependent increase in DCF fluorescence, becoming significant at 3.1 µg/cm^2^ and above. Furthermore, at the two intermediate concentrations of 3.1 and 6.3 µg/cm^2^, both fine and ultrafine PM induced significantly greater DCF fluorescence than did coarse PM. It was also observed that the great majority of the fluorescence was located in the supernatant, indicative of DCF leakage.

**FIG. 3.**
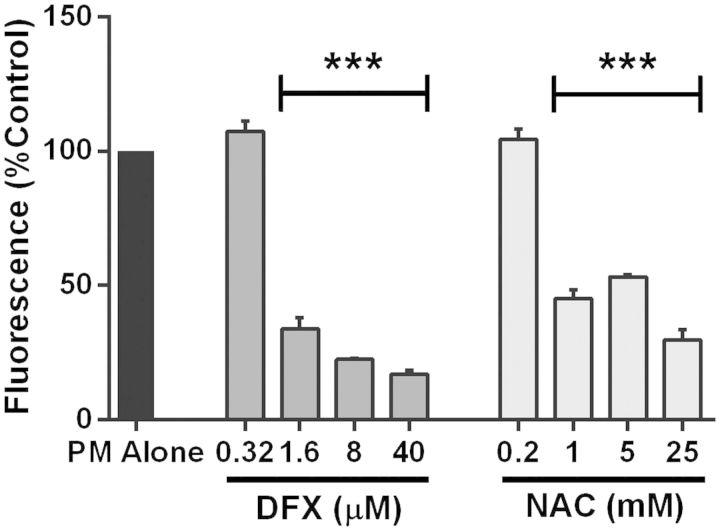
Modulation of reactive oxygen species (ROS) generation by desferrioxamine (DFX) and N-acetylcysteine (NAC). The effect of DFX and NAC on 2,7-dichlorofluorescein (DCF) fluorescence induced by ultrafine underground railway particulate matter (PM) was studied by challenging 16HBE monolayers with 3.1 µg/cm^2^ ultrafine PM in the presence of varying concentrations of DFX or NAC. DCF fluorescence was measured after 3 h incubation. Data were calculated as for Figure 2, followed by calculation of the percentage fluorescence in DFX/NAC-supplemented wells compared with DFX/NAC-free wells, all in duplicate. Mean ( ± SEM) fold increase in DCF fluorescence vs*.* control in absence of DFX and NAC was 10.2 ± 0.4. ***P < 0.001 vs*.* DFX/NAC-free culture, *n* = 3 for DFX experiments, *n* = 4 for NAC experiments.

Given its novel iron-rich nature and heightened ROS-generating ability, the ultrafine fraction was selected for further mechanistic studies using the iron chelator DFX and the free radical scavenger NAC. These experiments used monolayers of the bronchial epithelial cell line 16HBE due to the requirement for a large number of cells. In combination with 3.1 µg/cm^2^ ultrafine underground PM, DFX concentrations of 1.6 µM and 40 µM inhibited ultrafine PM-induced ROS generation to 33.8 ± 4.1% and 17.1 ± 1.4% of PM-only controls, respectively (*P* < 0.001), suggesting that ROS generation by ultrafine underground PM is due principally to the presence of iron (Fig. 3). Similarly, NAC concentrations of 1 mM and 25 mM inhibited DCF fluorescence to 45.2 ± 3.1% and 30.0 ± 3.8% of the PM-only control, respectively (*P* < 0.001), indicating that the majority of underground PM-induced DCF fluorescence was due to the presence of ROS.

##### Antioxidant gene expression in PBEC monolayers

**FIG. 4.**
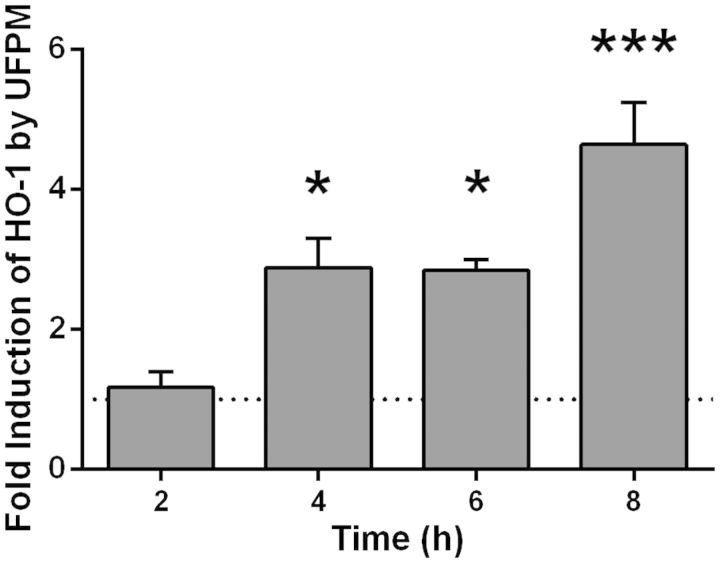
Changes in heme oxygenase-1 (HO-1) gene expression with time in primary bronchial epithelial cell (PBEC) monolayer cultures after challenge with ultrafine underground railway particulate matter (PM). Healthy donor PBEC monolayer cultures were challenged with 5.6 µg/cm^2^ ultrafine underground railway PM. At various time points, cells were lysed and gene expression determined by RT-qPCR. Data calculated by the ΔΔCt method, normalizing first to housekeeping gene (ubiquitin C [UBC]/glyceraldehyde 3-phosphate dehydrogenase [GAPDH]) expression for ΔCt, and then to expression of control (PM-free) wells for each timepoint for ΔΔCt. *P < 0.05, ***P < 0.001 vs*.* UF PM-free control at respective timepoint, *n* = 3 healthy donors.

Because cells may be able to defend against the effects of ROS by up-regulating antioxidant production, the expression of the antioxidant enzyme HO-1 was measured at various timepoints (Fig. 4). After 4 h exposure to 5.6 µg/cm^2^ ultrafine underground PM, HO-1 expression was increased by 2.9 ± 0.4-fold compared with control (*P* < 0.05), and this increased to 4.6 ± 0.6-fold by 8 h (*P* < 0.001), indicating activation of antioxidant mechanisms to counteract the presence of ROS.

##### IL-8 response of ALI cultures to underground railway PM

**FIG. 5.**
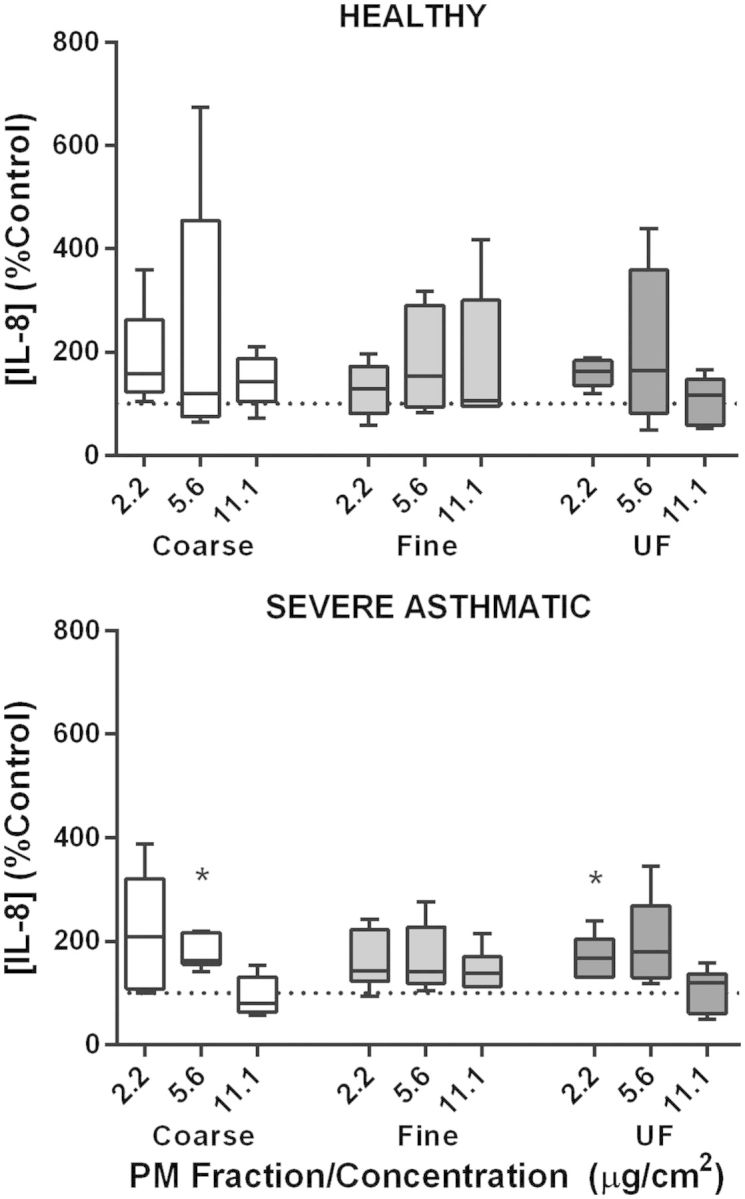
The effect of underground railway particulate matter (PM) on IL-8 release from healthy and severely asthmatic donor differentiated primary bronchial epithelial cell (PBEC) cultures at air-liquid interface (ALI). ALI cultures of PBECs from cells of healthy (top) or severely asthmatic (bottom) donors were exposed to varying concentrations of coarse, fine, or ultrafine underground railway PM for 24 h. Basolateral supernatant IL-8 concentration was analyzed by ELISA, with supernatants assayed in duplicate. IL-8 release is expressed as percentage of IL-8 release from cells incubated in control medium for 24 h. Median IL-8 concentration in controls was 7791 pg/ml, IQR 3051–13713 pg/ml (healthy) and 2866 pg/ml, IQR 1312–4419 pg/ml (severe asthmatic). Results presented as median with 25th and 75th percentile, whiskers represent 10th and 90th percentiles. *P < 0.05 vs. control, *n* = 5 healthy donors, 6 severely asthmatic donors.

Having characterized the basic responses to underground railway PM of PBEC monolayers, further work was aimed at assessing responses in ALI cultures of PBECs. After 24 h exposure to underground PM, there was no significant change in epithelial barrier permeability of healthy or severely asthmatic donor ALI cultures, as assessed by 4 kDa FITC-dextran passage (Supplementary fig. S2). There was a significant induction of IL-8 release from severely asthmatic donor ALI cultures with 5.6 µg/cm^2^ coarse underground PM and 2.2 µg/cm^2^ ultrafine PM (Fig. 5). However, there was no concentration dependence, and the greatest significant increase (median 168% with 2.2 µg/cm^2^ ultrafine PM) was considerably lower than seen in monolayer cultures, suggesting that the phenotypic difference between ALI cultures and monolayer cultures affects IL-8 responses and/or that the mucous layer retards the movement or effects of particles, protecting the cells. Furthermore, microscopic examination of cell cultures indicated that there was no evidence of apoptotic or necrotic cell death in these cultures, which might otherwise explain this lack of response (Supplementary fig. S3).

##### Cellular antioxidant responses to ultrafine PM

**FIG. 6.**
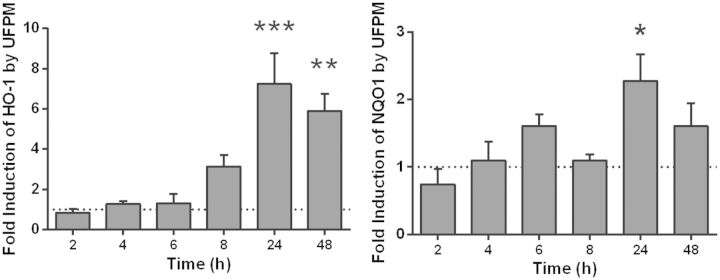
Changes in antioxidant gene expression with time in air-liquid interface (ALI) cultures after challenge with ultrafine underground railway particulate matter (PM). Healthy donor primary bronchial epithelial cell (PBEC) ALI cultures were challenged with 5.6 µg/cm^2^ ultrafine underground railway PM. At various time points, cells were lysed and gene expression determined by RT-qPCR. Data calculated by the ΔΔCt method, normalizing first to housekeeping gene (ubiquitin C [UBC]/glyceraldehyde 3-phosphate dehydrogenase [GAPDH]) expression for ΔCt, and then to expression of control (PM-free) wells for each timepoint for ΔΔCt. Data presented as mean ± SEM. *P < 0.05, **P < 0.01, ***P < 0.001 vs*.* UF PM-free control at respective timepoint, *n* = 3 healthy donors.

**FIG. 7.**
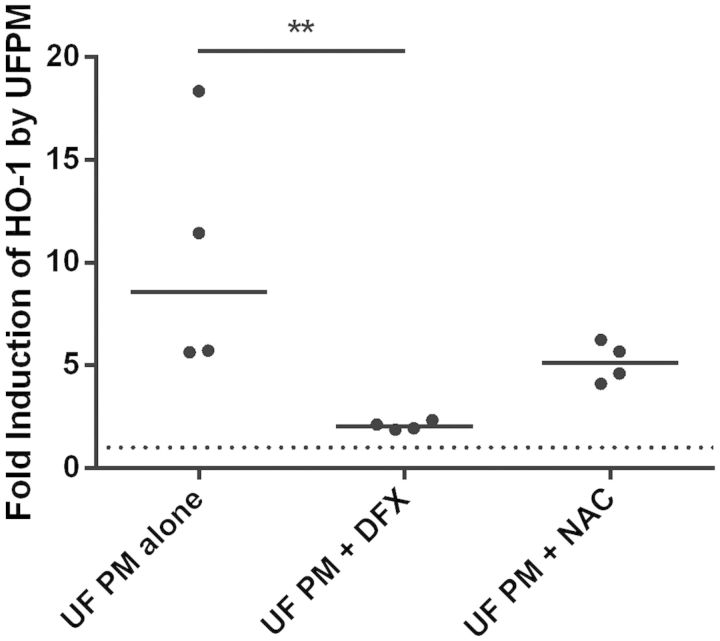
The effect of an iron chelator (DFX) and a free radical scavenger (N-acetylcysteine [NAC]) on heme oxygenase-1 (HO-1) induction by ultrafine underground particulate matter (PM). Healthy donor air-liquid interface (ALI) cultures were incubated for 24 h with 5.6 µg/cm^2^ ultrafine underground PM, after which time RNA was harvested. HO-1 expression was determined by RT-qPCR in duplicate and normalized to PM-free incubations (with DFX, NAC, or neither) by the ΔΔCt method. Individual data points are shown, line represents median value. **P < 0.01, *n* = 4 healthy donors.

To determine whether ALI cultures exhibited a similarly diminished antioxidant response compared with PBECs, ALI cultures of healthy donor cells were challenged with 5.6 µg/cm^2^ ultrafine PM for varying time periods, following which expression levels of two antioxidant genes were quantified (Fig. 6). Over the period of challenge with ultrafine PM, heme oxygenase-1 (HO-1) expression rose steadily to a peak at 24 h, a significant fold increase of 7.2 ± 1.5 over control levels (*P* < 0.001) but a slower response than had been observed in monolayers. By 48 h, levels had begun to fall, although they were still significantly elevated compared with the control. For NQO1, a similar trend was observed, reaching a peak at 24 h (*P* < 0.05). To study this response further, healthy donor ALI cultures were challenged for 24 h with 5.6 µg/cm^2^ ultrafine PM in the presence of either 200 µM DFX or 20 mM NAC (Fig. 7). Exposure to 5.6 µg/cm^2^ ultrafine underground railway PM resulted in a median 8.6-fold increase in HO-1 expression (IQR: 5.7–17). In the presence of 200 µM DFX, the PM-induced response was reduced to a median 2.0-fold induction of HO-1 (IQR: 1.9–2.3, *P* < 0.01). NAC tended to diminish HO-1 induction by ultrafine underground PM (nonsignificant). These data suggest that the increase in HO-1 elicited by ultrafine underground PM is predominantly due to the iron content of the PM.

##### Uptake of underground railway PM by ALI-cultured PBECs

**FIG. 8.**
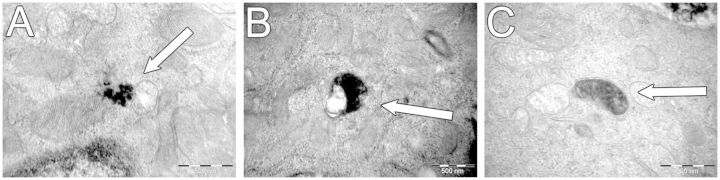
Intracellular localization of underground railway particulate matter (PM) in primary bronchial epithelial cell (PBEC) air-liquid interface (ALI) cultures after 24 h incubation. Healthy donor ALI cultures were incubated with coarse, fine, or ultrafine underground railway PM for 24 h, after which time they were washed, fixed, and embedded for transmission electron microscopy. At least eight ultrafine sections were viewed for each of two areas per culture, with areas at least 100 µm apart. No obvious differences were noted in quantity or localization of between PM size fractions. PM is shown free in the cytosol in proximity to a mitochondrion (A, treated with 3 µg/cm^2^ coarse PM), within a membrane-bound vesicle (B, treated with 12 µg/cm^2^ ultrafine PM), and apparently within a mitochondrion (C, treated with 3 µg/cm^2^ coarse PM). No PM was observed within cell nuclei. Scale bar represents 500 nm.

To determine whether PM could penetrate the mucus barrier and access cells, ALI cultures were exposed to underground PM at concentrations of 3 and 12 µg/cm^2^ for 24 h, following which they were examined by transmission electron microscopy (TEM) (Fig. 8). The results indicated that particles of the coarse, fine, and ultrafine fractions of underground railway PM are able to enter epithelial cells, although intracellular particles were generally of a geometric diameter <1 µm. PM was observed free within the cytosol, in membrane-bound vesicles, and in structures resembling mitochondria, although no PM was observed within nuclei. The presence of intracellular PM did not, however, appear to induce any morphological differences in cells, and most cells were PM-free. This intracellular PM was confirmed as underground PM by energy-dispersive x-ray analysis to detect iron (Supplementary fig. S4).

## DISCUSSION

This study examined the responses of bronchial epithelial cells to size-fractionated, iron-rich underground railway PM. The results indicate that underground PM is able to generate ROS, with the induction of inflammatory and anti-oxidant responses in PBEC cultures, which may be diminished but not abolished by the presence of a mucous layer.

Although monolayers are not an ideal approximation of the cellular configuration of the airway epithelium because they contain only one cell type, do not express apicobasal polarity, and do not possess ciliated or mucus secreting cells, they are frequently used for basic concentration-response characterization. PM concentration-dependent IL-8 release was observed, with the magnitude of IL-8 release being similar to that previously observed from the alveolar epithelial cell line A549 exposed to London Underground PM_2.5_, albeit over only an 8 h exposure, also without significant LDH release at particle concentration equivalent to those in this study (Seaton *et al.*, 2005). Asthmatic donor PBECs have been seen to be more susceptible to the effects of hydrogen peroxide (Bucchieri *et al.*, 2002), but this was at concentrations sufficient to induce apoptosis, and the same may not apply to relatively insoluble PM. Indeed, LDH assay of PM-exposed monolayers showed no significant cell death in response to PM. Furthermore, it may be that features of asthma which heighten sensitivity to airborne PM are not recapitulated in PBEC monocultures or that mechanisms other than oxidative stress are involved in induction of IL-8 release by metal-rich PM (Steenhof *et al.*, 2013). It is unlikely that the slightly suppressed responses of the PBECs from asthmatic donors was due to carry-over of corticosteroids, given the short plasma half-life of inhaled corticosteroids and steroid-receptor complexes (Derendorf *et al.*, 1998; Johnson, 1998) and the duration of culture prior to challenge. The marked IL-8 response to TNF-α suggested that the cells did not mount a maximal response to underground railway PM. As is evident in Supplementary table S1, there was significant donor heterogeneity, including smoking status. However, additional analysis involving grouping by smoking status rather than asthma status showed no significant effect of smoking status on IL-8 response to PM (data not shown).

Because ROS are considered to be a major intermediate through which airborne PM exerts its effects, ROS generation was measured using the dye DCF, which indicated that underground railway PM generates ROS in a concentration-, size-, and iron-dependent manner. In light of the apparent leakage of DCF, these data do not necessarily indicate that ROS generation is intracellular, but they do suggest that PM is able to generate ROS in the pericellular milieu. Leakage of DCF following replacement of the extracellular medium has been noted previously (Royall and Ischiropoulos, 1993), although this may depend on cell type (Swift and Sarvazyan, 2000). It has been suggested that leakage may be due to differences in intracellular esterase partitioning that affects cleavage of the diacetate groups and thus intracellular retention of DCF (Robinson *et al.*, 1988). Furthermore, it has been suggested that oxidation of DCF is not simply indicative of general oxidative stress, since it is only oxidized by the hydroxyl radical, thus requiring the presence of iron or directly by cytochrome c (Karlsson *et al.*, 2010). PM surface area/volume ratio is an important determinant of PM reactivity because only the surface of the particle is able to interact with the surrounding milieu, but chemical composition is also important. In ambient PM, metals may be found in greater abundance in the coarse fraction compared with the ultrafine fraction (Li *et al.*, 2003), which comprises both directly emitted primary particles and the products of secondary reactions between gases and carbon (Kim *et al.*, 2002). However, in the present underground PM samples, the ultrafine fraction was at least as rich in iron and other transition metals. The combination of metal-rich composition and high surface area/volume ratio suggested that ultrafine underground railway PM may be the most potent ROS generator of the three fractions, and this was confirmed by the experimental data, which also used DFX and NAC to demonstrate that this ROS generation is mediated by iron-generated free radicals.

Interestingly, the release of IL-8 from ALI cultures in response to underground PM was neither PM concentration-dependent nor of the same magnitude seen in monolayers. Although asthmatic donors appeared to be more sensitive to underground PM in terms of IL-8 release, this was a modest effect and not significantly different to healthy donor cultures. Indeed, given their tendency to exhibit a denuded basal epithelium (Montefort *et al.*, 1992), it may be that susceptible areas of asthmatic airways are better represented by monolayer cultures. The most likely explanation for the relatively smaller effect in ALI cultures may be the presence of a mucous layer. In ALI culture experiments, centrifugation of the apical supernatant yielded a gray pellet, likely containing PM, whereas PM was still visible under light microscope on/in monolayer cultures but not ALI cultures, after washing. The observation that iron in underground PM is generally found in the form of relatively water-insoluble oxides (Karlsson *et al.*, 2008; Querol *et al.*, 2012) further reduces the potential for PM components being able to penetrate the mucous barrier.

Given the ability of underground PM to generate ROS, the antioxidant response of the cells to the ultrafine fraction, which combined a high metal content and high surface area/volume ratio, was studied. Both HO-1 and NQO1 have previously been seen to be up-regulated by PM (Bachoual *et al.*, 2007; Billet *et al.*, 2007). Herein, 24-h exposure to ultrafine PM resulted in HO-1 upregulation, which was inhibited by DFX and tended to decrease with NAC. Furthermore, the retardation of this process in ALI cultures compared with monolayer cultures suggests that the mucus may be restricting either the movement of particles toward the cells or generation of ROS. Alternatively, there may be iron-independent ROS generation. For example, organic extracts of diesel exhaust particles are able to induce expression of HO-1 and NQO1 (Baulig *et al.*, 2003). One drawback of this study was that the organic components of underground railway PM were not analyzed, although elemental and organic carbon concentrations of PM collected during a different sampling campaign at the same station have been published previously (Strak *et al.*, 2011).

The reduced inflammatory response in ALI cultures may indicate that antioxidant defenses or the presence of a mucous layer diminishes oxidative stress to a level below that at which proinflammatory effects are seen. To better understand the underground PM-cell interactions, ALI cultures exposed to underground PM for 24 h were examined for the presence of intracellular PM. Published results are inconsistent, suggesting that uptake is neither necessarily dependent on PM concentration, nor linked to cytotoxicity (Belade *et al.*, 2012), and may vary between cell types (Schaudien *et al.*, 2012). In this study, cytosolic, vesicular, and mitochondrial PM, but not nuclear PM, was observed, and all PM-containing cells appeared intact. Similarly, other studies have noted that intracellular PM is generally found within vesicles or free within the cytosol in 16HBE and A549 cells and almost never in the nucleus (Belade *et al.*, 2012; Calcabrini *et al.*, 2004; Konczol *et al.*, 2011). Ultrafine particles have been observed in mitochondria of RAW 264.7 macrophages and BEAS-2B cells (Li *et al.*, 2003). Mitochondrial damage, which may be a consequence of ROS generation by PM constituents (Karlsson *et al.*, 2008; Upadhyay *et al.*, 2003), may result in reduced mitochondrial membrane potential and further increased ROS generation due to electron transport chain uncoupling (Freyre-Fonseca *et al.*, 2011). The potential effects of intracellular accumulation of underground PM require more work, especially with respect to the ultrafine fraction.

It is difficult to accurately measure *in vivo* deposition of particles within the airways. It has been proposed that, at a PM concentration of 100 µg/m^3^ (ie, lower than in underground railways), average tracheobronchial deposition of PM is likely to be in the ng/cm^2^ range but that nonuniform exposure such as at bifurcation points, with raised breathing rate, increased oral breathing, and altered airway geometry may raise localized deposition to over 80 µg/cm^2^ (Phalen *et al.*, 2006). Considering that underground PM concentrations are generally higher than 100 µg/m^3^, this suggests that PM concentrations used in this study are well within the possible real life *in vivo* range.

This study suggests that short-term exposure to PM containing relatively high concentrations of iron, copper, and other transition metals, from underground railways and potentially other transport sources such as tramway and overground railway systems, can result in acute effects on airway epithelial cells. However, as discussed in the Introduction, there is a paucity of evidence in the literature for effects with either acute or chronic exposure in humans. One reason for this may be that the induction of airway antioxidant expression, along with macrophage and mucociliary particle clearance mechanisms, might be able to mitigate the presence and effects of the PM. Conversely, it is possible that individuals with certain diseases may have less of a “buffering” capacity, and therefore be at greater risk of the effects of such PM (Maynard, 2013). In this regard, it is interesting that *in vivo* studies of acute effects of underground railway PM exposure have generally focused on healthy or mildly asthmatic individuals. The question of occupational risks following chronic exposure is considerably more complicated, since it involves matters of repeat exposures, bioavailability and biopersistence of particles, and also the potential for injury to other compartments, principally the cardiovascular system. There is a large amount of evidence that chronic exposure to airborne PM is detrimental to health and a major cause of morbidity and mortality (Brunekreef and Holgate, 2002; Lim *et al.*, 2012), but the specific effects of long-term exposure to underground PM require further study.

In summary, coarse, fine, and ultrafine fractions of underground railway PM are able to induce an acute proinflammatory response from PBEC monolayers but less so in ALI cultures. Generation of ROS by underground railway PM was size- and iron dependent, with iron in the ultrafine particles being responsible for inducing an antioxidant response. Furthermore, PM is able to penetrate the mucous layer and enter the epithelial cells, being found free in the cytosol and in structures which appear to be mitochondria, although it also appears that the presence of mucus may considerably attenuate some of the effects of such PM. Given the unusually iron-rich nature of the ultrafine fraction, and its potency in generating ROS, further work is required to determine whether the ultrafine fraction of underground railway PM has the potential to damage health, either by effects on the respiratory system which spill over into a systemic inflammatory response, or by translocation to the systemic circulation (Brook *et al.*, 2010).

## SUPPLEMENTARY DATA

Supplementary data are available online at http://toxsci.oxfordjournals.org/.

Supplementary Data
