# Supplementary material for: The Effects on Bronchial Epithelial Mucociliary Cultures of Coarse, Fine, and Ultrafine Particulate Matter From an Underground Railway Station
Source: Toxicol Sci. 2015 Feb 10;145(1):98–107. doi: 10.1093/toxsci/kfv034 (PMC4408962; doi:10.1093/toxsci/kfv034)
Supplement: Supplementary Data [file supp_145_1_98__index.html]

The Effects on Bronchial Epithelial Mucociliary Cultures of Coarse, Fine, and Ultrafine Particulate Matter From an Underground Railway Station — The Effects on Bronchial Epithelial Mucociliary Cultures of Coarse, Fine, and Ultrafine Particulate Matter From an Underground Railway Station — The Effects on Bronchial Epithelial Mucociliary Cultures of Coarse, Fine, and Ultrafine Particulate Matter From an Underground Railway Station — Supplementary Data 

# The Effects on Bronchial Epithelial Mucociliary Cultures of Coarse, Fine, and Ultrafine Particulate Matter From an Underground Railway Station

## Supplementary Data

files

**Files in this Data Supplement:**

- Supplementary Data - tif file
- Supplementary Data - tif file
- Supplementary Data - tif file
- Supplementary Data - tif file
