# Supplementary figures and images for: The Effects on Bronchial Epithelial Mucociliary Cultures of Coarse, Fine, and Ultrafine Particulate Matter From an Underground Railway Station
Source: Toxicol Sci. 2015 Feb 10;145(1):98–107. doi: 10.1093/toxsci/kfv034 (PMC4408962; doi:10.1093/toxsci/kfv034)

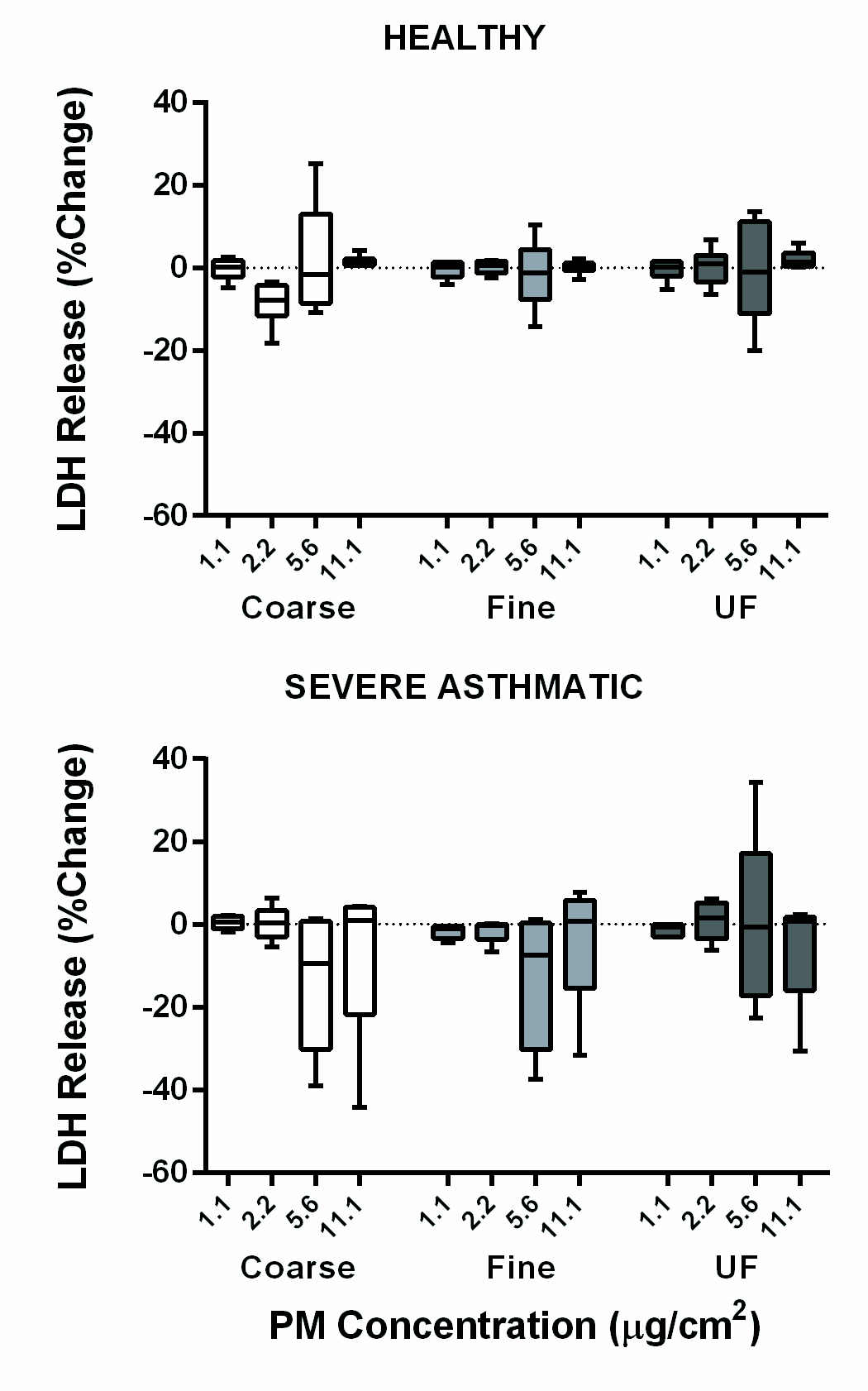

Supplement: Supplementary Data [file supp_kfv034_toxsci-14-0825-File010.tif]

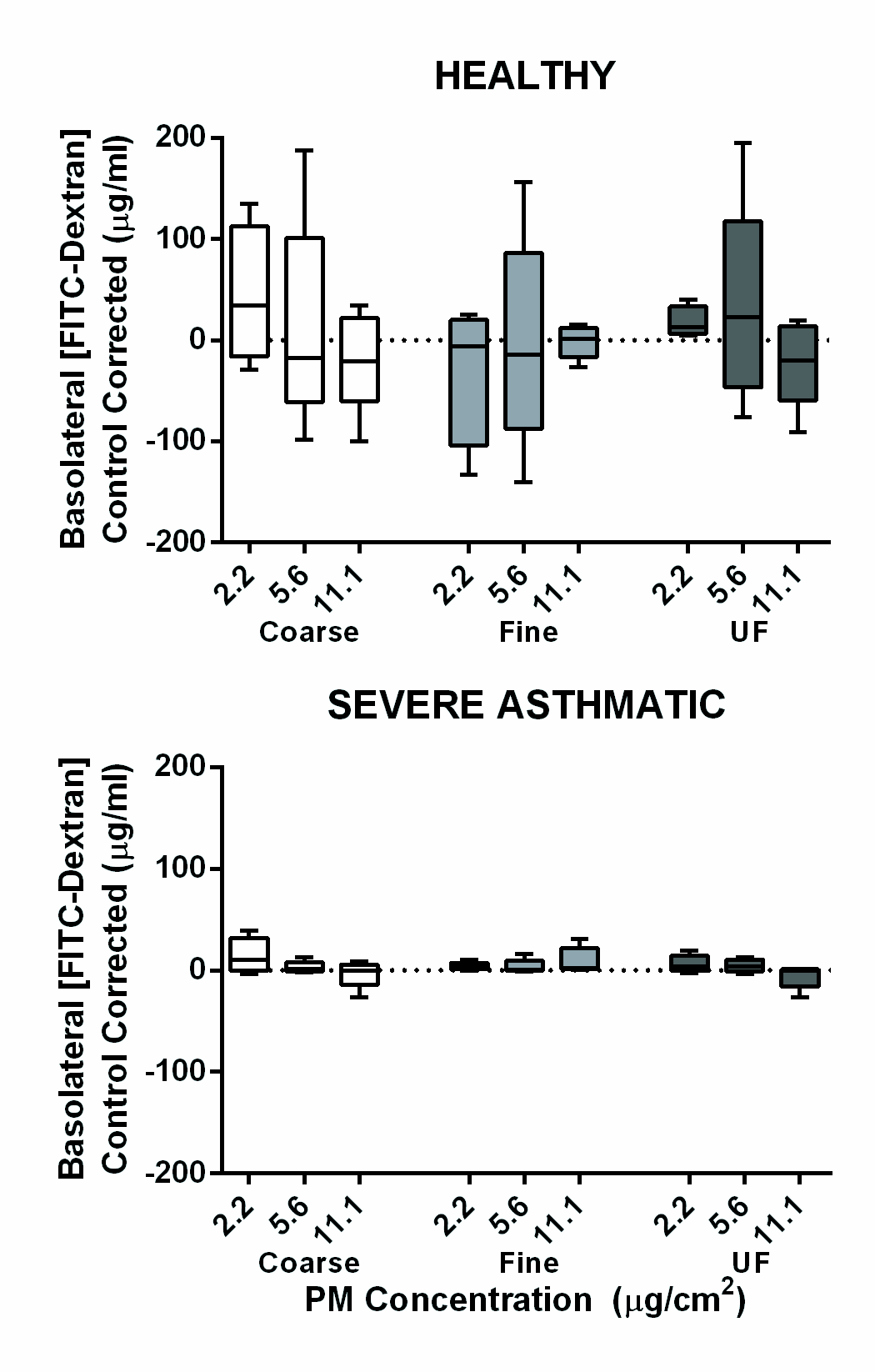

Supplement: Supplementary Data [file supp_kfv034_toxsci-14-0825-File011.tif]

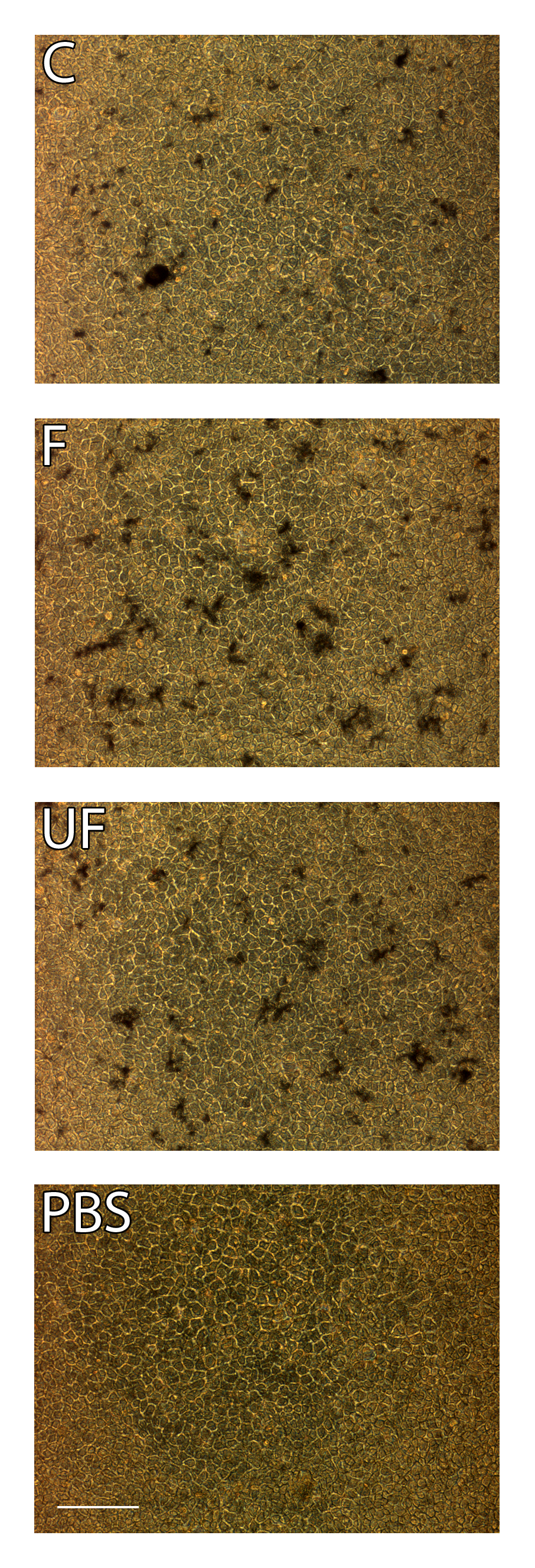

Supplement: Supplementary Data [file supp_kfv034_toxsci-14-0825-File012.tif]

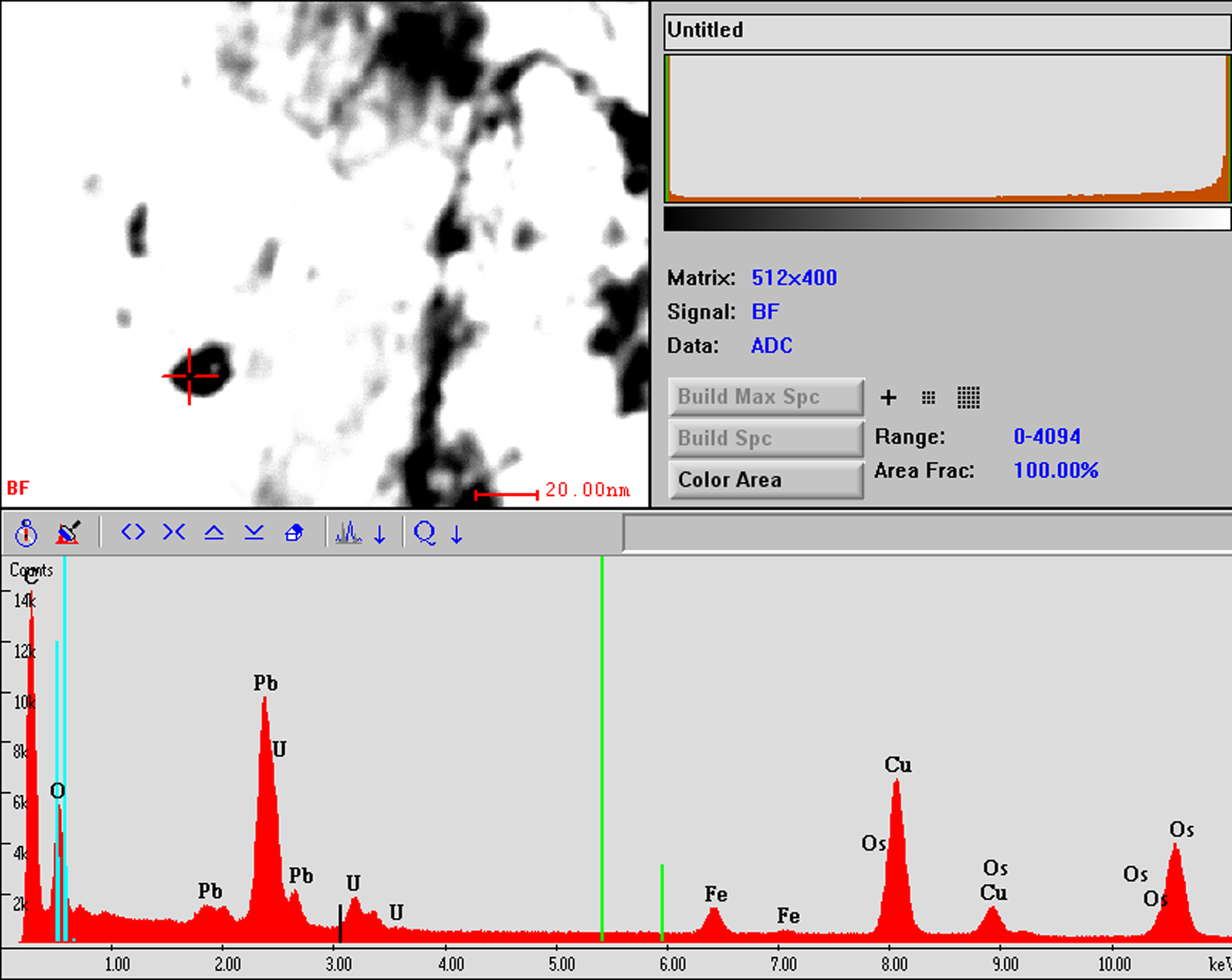

Supplement: Supplementary Data [file supp_kfv034_toxsci-14-0825-File013.tif]
